# The Daily Expression of ABCC4 at the BCSFB Affects the Transport of Its Substrate Methotrexate

**DOI:** 10.3390/ijms23052443

**Published:** 2022-02-23

**Authors:** André Furtado, Rafael Mineiro, Ana Catarina Duarte, Isabel Gonçalves, Cecília R. Santos, Telma Quintela

**Affiliations:** 1 CICS-UBI—Health Sciences Research Centre, University of Beira Interior, Av. Infante D. Henrique, 6200-506 Covilhã, Portugal; andre.furtado@ubi.pt (A.F.); rafael.m.mineiro@ubi.pt (R.M.); anacduarte28@hotmail.com (A.C.D.); igoncalves@fcsaude.ubi.pt (I.G.); csantos@fcsaude.ubi.pt (C.R.S.); 2 UDI-IPG—Unidade de Investigação para o Desenvolvimento do Interior, Instituto Politécnico da Guarda, 6300-559 Guarda, Portugal

**Keywords:** choroid plexus, circadian rhythms, membrane transporters, sex hormones

## Abstract

The choroid plexuses (CPs), located in the brain ventricles, form an interface between the blood and the cerebrospinal fluid named the blood-cerebrospinal barrier, which, by the presence of tight junctions, detoxification enzymes, and membrane transporters, limits the traffic of molecules into the central nervous system. It has already been shown that sex hormones regulate several CP functions, including the oscillations of its clock genes. However, it is less explored how the circadian rhythm regulates CP functions. This study aimed to evaluate the impact of sex hormones and circadian rhythms on the function of CP membrane transporters. The 24 h transcription profiles of the membrane transporters rAbca1, rAbcb1, rAbcc1, rAbcc4, rAbcg2, rAbcg4, and rOat3 were characterized in the CPs of intact male, intact female, sham-operated female, and gonadectomized rats. We found that rAbcc1 is expressed in a circadian way in the CPs of intact male rats, rAbcg2 in the CPs of intact female rats, and both rAbcc4 and rOat3 mRNA levels were expressed in a circadian way in the CPs of intact male and female rats. Next, using an in vitro model of the human blood–cerebrospinal fluid barrier, we also found that methotrexate (MTX) is transported in a circadian way across this barrier. The circadian pattern of Abcc4 found in the human CP epithelial papilloma cells might be partially responsible for MTX circadian transport across the basal membrane of CP epithelial cells.

## 1. Introduction

To adapt to the environmental changes, living organisms have developed circadian rhythms, which, ultimately, correspond to daily oscillations in biological processes [1,2,3]. The mammalian circadian system is conceptualized in a hierarchical way, in which the suprachiasmatic nucleus (SCN) of the hypothalamus operates as the master clock. The SCN is responsible for receiving light information via the optic nerve and synchronizing the remaining clocks in the body [4]. At the cellular level, Circadian Locomotor Output Cycles Kaput (CLOCK) and Brain and Muscle ARNT-Like 1 (BMAL1) proteins form a complex that promotes the transcription of many genes, including the negative regulators Period (*Per1*, *Per2*, and *Per3*) and Cryptochrome (*Cry1* and *Cry2*). Subsequently, PER and CRY proteins interact with CLOCK–BMAL1 complexes, repressing their own transcription and the transcription of many clock-controlled genes [5].

The circadian system has an impact on the disposal and action of drugs, determining the efficacy and toxicity of several therapeutic agents [6,7]. This evidence lends support to the idea that daily variations of drug-metabolizing enzymes and transport systems may interfere with drug pharmacokinetics, particularly in the absorption, distribution, metabolism, and elimination mechanisms [8,9,10]. Consequently, some authors have raised interest in studying and understanding the molecular pathways involved in the circadian control of detoxifying enzymes and specific influx/efflux transporters to enhance therapeutic efficacy and minimize side effects [8,9,11,12] by adjusting drug administration schedules to follow circadian rhythms.

The choroid plexuses (CPs) are part of the ventricular system of the brain and are located in each of the four brain ventricles. Each CP is composed of a monolayer of cuboidal epithelial cells that lay in a basement membrane. Below, in the stroma, resides a network of fenestrated capillaries surrounded by connective tissue and immune system cells [13]. The primary role of CPs is to produce the cerebrospinal fluid (CSF), but the CPs also form an important barrier between the blood and the CSF, the blood–CSF barrier (BCSFB) [14], which has been overlooked for years. The presence of tight junctions, detoxification enzymes, and membrane transporters in the epithelial cells enable the CP epithelia to control the traffic of molecules, including therapeutic agents, into the central nervous system (CNS) [15]. Another important function of the CP is that it holds a circadian clock composed of clock genes that are under circadian regulation, with pronounced differences between male and female rats [16,17] and overall susceptibility to sex hormones.

ATP-binding cassette (ABC) and solute carrier (SLC) transporters are two families of membrane proteins responsible for the extrusion of molecules out of the cells, and they are widely known by their contribution to pharmacoresistance in many cell types, in which they are able to recognize and extrude a vast array of therapeutic drugs [18]. At the BCSFB, previous literature described the presence of several multispecific ABC (ABCB1, ABCG2, ABCC1, and ABCC4) and SLC (OAT3) transporters [19]. The selective expression of these transporters at the basolateral membrane (ABCC4; ABCC1) are thought to be involved in the extrusion of molecules back to the bloodstream, preventing their entry into the CNS [20,21]. At the apical membrane, ABCB1 and ABCG2 transporters transfer molecules from the epithelial cells to the CSF.

The presence of a sex-dependent circadian oscillator in the CP and the involvement of this tissue in the traffic of molecules from the blood to the CSF and, consequently, to the brain because of the presence of membrane transporters holds promise for the concertation of the circadian pattern of efflux transporters with the best timing for drug administration. For that, a deep knowledge of how the circadian rhythms tune the expression and function of CP membrane transporters is required. Thus, in the present study, we explored the sex-related differences of the daily oscillation of ABC and SLC transporters in rat CPs and investigated the relevance of Abcc4 circadian expression in the transport of methotrexate (MTX) across the BCSFB.

## 2. Results

### 2.1. Sex-Dependent Daily Oscillations of Membrane Transporters in Rat Choroid Plexus

To analyze whether the CP membrane transporters exhibit a gender-specific daily oscillation, the temporal expression profile of several transporters was assessed by RT-qPCR in the CPs of male and female rats.

**Figure 1:**
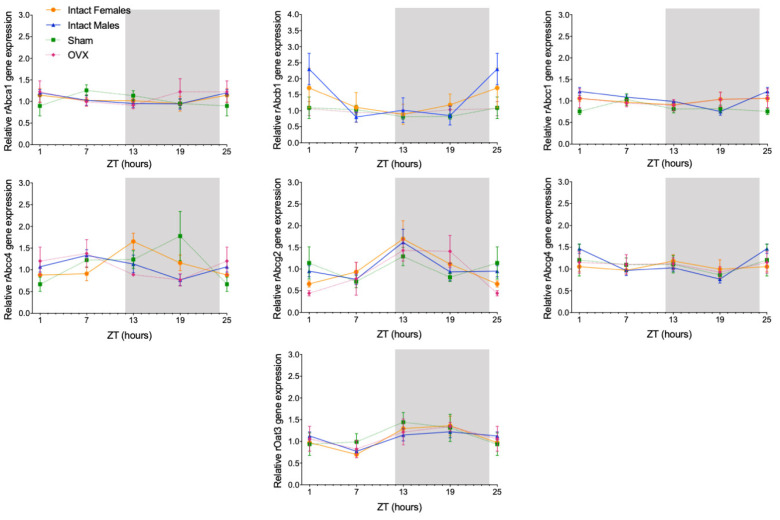
Circadian transcription profiles of membrane transporters in the CPs of intact males, intact females, ovariectomized (OVX), and sham-operated female rats (Sham). rAbca1, rAbcb1, rAbcc1, rAbcc4, rAbcg2, rAbcg4, and rOat3 mRNA circadian expression was analyzed. White and grey backgrounds represent the day and night periods, respectively. Panel shows the mean ± SEM transcript levels (*n* = 3–6), and data from ZT1 and ZT25 are double plotted.

**Figure 2:**
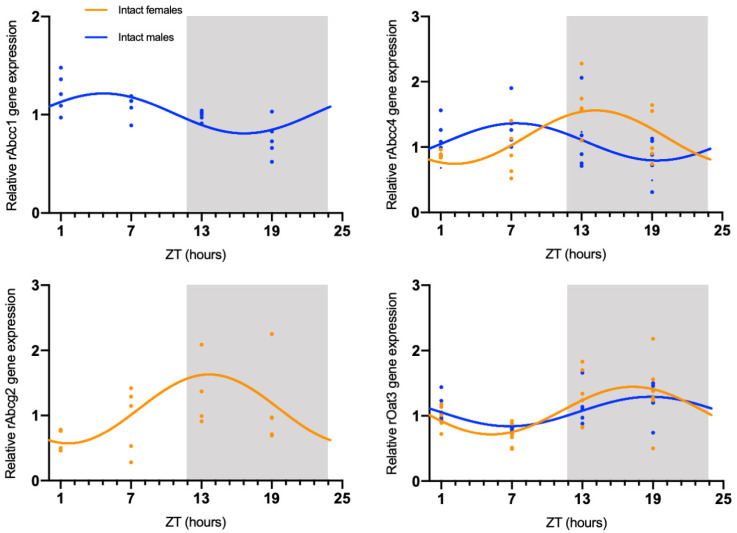
CircWave analysis of membrane transporters rAbcc1, rAbcc4, rAbcg2, and rOat3 mRNA circadian expression in the CPs of intact male and female rats. Absence of the CircWave curve indicates absence of significant rhythmicity as analyzed by CircWave. White and grey backgrounds represent the day and night periods, respectively.

The circadian mRNA expression profiles were obtained for rAbca1, rAbcb1, rAbcc1, rAbcc4, rAbcg2, rAbcg4, and rOat3 (Figure 1). rAbca1, rAbcb1, and rAbcg4 mRNA expression did not show significant daily oscillation (Figure 2, Table 1). On the contrary, Abcc1 mRNA expression in male rats showed a significant daily oscillation (CircWave, *p* < 0.05), with a peak during the light phase between ZT4 and ZT5 (Figure 2, Table 1). The mRNA levels of Abcc4 also showed a significant daily oscillation, but in this case in both intact male and female rats (CircWave, *p* < 0.05), with a peak level around ZT14 and ZT7, respectively. A significant daily oscillation of rAbcg2 was also verified in intact female rats (CircWave, *p* < 0.05), with a peak level around ZT14. Lastly, rOat3 mRNA levels in intact male and female rats exhibited a significant daily oscillation (CircWave, *p* < 0.05), with a peak of expression during the first half of the dark phase between ZT17 and ZT19 (Figure 2, Table 1).

### 2.2. hAbcc4 and hAbcg2 mRNA Circadian Expression in the HIBCPP Cell Line

**Figure 3:**
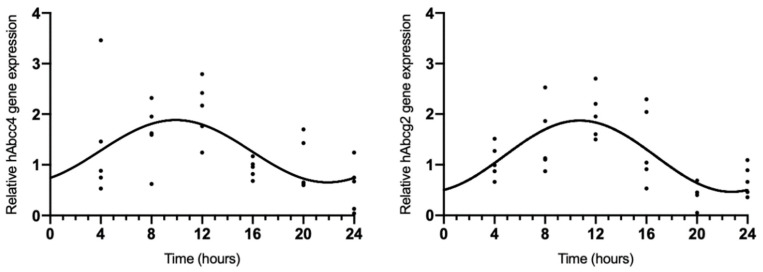
CircWave analysis of hAbcc4 and hAbcg2 expression profile in the HIBCPP cells. The represented curve indicates a statistically significant rhythm (CircWave, *p* < 0.05).

Because rAbcc4 showed daily oscillations in intact male and female rats and rAbcg2 showed daily oscillations in intact female rats, we also assessed the temporal expression of hAbcc4 and hAbcg2 mRNA in the human HIBCCP cell line. The results showed circadian variation (CircWave, *p* < 0.05) with a peak around 10 h after synchronization (Figure 3, Table 2).

### 2.3. Circadian Oscillations in MTX Transport across BCSFB

**Figure 4:**
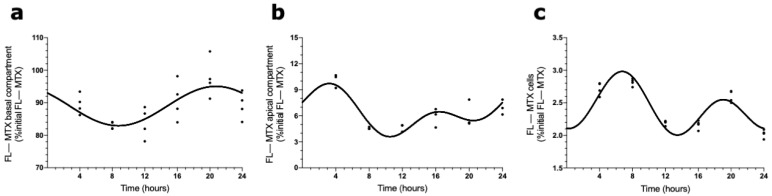
FL–MTX transport across the BCSFB. CircWave analysis of FL–MTX levels from basal (**a**), apical (**b**), and intracellular (**c**) compartments. The represented curves indicate a statistically significant rhythm (CircWave, *p* < 0.05).

The presence of a circadian pattern of Abcc4 in intact male and female rat CPs led us to examine if the transport of MTX across the BCSFB is circadian-dependent. According to the results shown in Figure 4, the FL–MTX concentration in the basal compartment oscillated (CircWave, *p* < 0.05), with a peak at 21 h after synchronization (Figure 4a). In the apical compartment, a significant oscillation in MTX concentration (CircWave, *p* < 0.05) was also observed, with a well-sustained peak around 3 h after synchronization (Figure 4b). Finally, in the intracellular compartment, the FL–MTX concentration also displayed circadian variation (CircWave, *p* < 0.05), with a pronounced peak at 7 h after synchronization (Figure 4c).

### 2.4. Effects of ABCC4 and ABCG2 Inhibition in the MTX Circadian Transport across BCSFB

**Figure 5:**
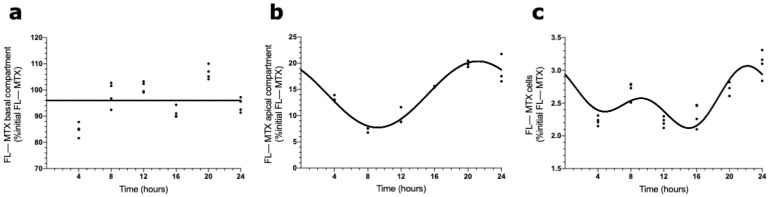
Effects of ABCC4 inhibition in the MTX circadian transport across the BCSFB. CircWave analysis of FL–MTX levels from basal (**a**), apical (**b**), and intracellular (**c**) compartments after the inhibition of ABCC4. The represented curves indicate a statistically significant rhythm (CircWave, *p* < 0.05).

To evaluate the involvement of ABCC4 in the circadian transport of FL–MTX across the BCSFB, an analogous in vitro transport assay was carried out using a selective ABCC4 inhibitor (ceefourin). We found that in the basal compartment, the MTX concentration did not show a circadian variation (Figure 5a). On the contrary, in the apical compartment, the FL–MTX concentration oscillated (CircWave, *p* < 0.05), with a peak around 21 h after synchronization (Figure 5b). At the intracellular compartment, a circadian variation was also observed in FL–MTX concentration (CircWave, *p* < 0.05), with a peak at 22 h (Figure 5c).

**Figure 6:**
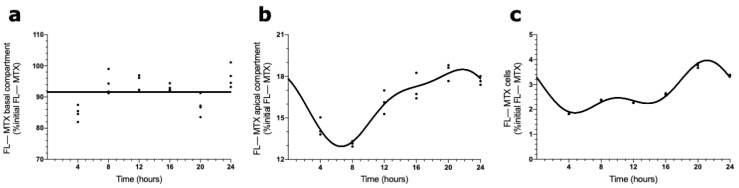
Effects of ABCG2 inhibition in FL–MTX circadian transport across the BCSFB. CircWave analysis of FL–MTX levels from basal (**a**), apical (**b**), and intracellular (**c**) compartments after the inhibition of ABCG2. The represented curves indicate a statistically significant rhythm (CircWave, *p* < 0.05).

ABCC4 is not the only membrane transporter involved in the brain efflux of MTX. For this reason, we assessed the influence of an apical transporter, ABCG2, in MTX transport across BCSFB using its specific inhibitor. ABCG2 is localized in the apical membrane of CPEC and revealed a circadian pattern in the CPs of intact females. In the basal compartment, as in the previous assay, no circadian variation was reported in the FL–MTX concentration (Figure 6a). In the apical compartment, the FL–MTX concentration oscillated (CircWave, *p* < 0.05), with a well-sustained peak at 20 h after synchronization (Figure 6b). Finally, in the intracellular compartment, the FL–MTX concentration also oscillated (CircWave, *p* < 0.05), and a peak can be observed between 21 and 22 h after synchronization (Figure 6c).

## 3. Discussion

The CP limits the passage of molecules from the blood to the CSF and, consequently, to the brain. This is partially ensured by transporter proteins located in the CPEC membrane. Thus, if the activity of a given transporter is subjected to circadian oscillations, drug administration of its known substrates must be scheduled according to those biological rhythms to improve drug delivery and the efficacy of the therapeutic agent. It is thus crucial to study the circadian rhythmicity of drug transporters and the associated molecular mechanisms.

In the present study, we showed that the rAbca1, rAbcb1, and rAbcg4 expression is not rhythmic in rat CPs. By contrast, rAbcc1 showed circadian rhythmicity in intact males, rAbcg2 in intact females, and rAbcc4 and rOat3 in both intact male and female rats. This is the first study addressing the circadian expression of any of these transporters in a brain barrier, but some studies show that ABC transporters are subject to circadian regulation in other tissues. The circadian expression of Abcc1 was studied in the Caco-2 cell line (derived from human colon adenocarcinoma) with a peak between the 6th and the 12th hour after synchronization [22]. Conversely, no rhythmicity was seen in the expression of Abcc1 in the jejunal mucosa of male rats [23], which is not in accordance with our results. In PARbZip transcription factor knockout mice, Abcc4 expression was reduced in the kidney, suggesting that Abcc4 is a clock-controlled gene [24]. The Abcb1 and Abcb4 genes contain a D-box response element [25,26], and an analysis of the 2000 bp upstream of the transcription start site of the human Abcc4 gene with the JASPAR CORE 2018 Vertebrates library revealed three putative DBP binding sites; therefore, its expression may be directly regulated by the PARbZip transcription factors. Abcc4 is also regulated by three xenobiotic receptors—constitutive androstane receptor (CAR), aryl hydrocarbon receptor (AhR), and pregnane X receptor (PXR) [27,28,29,30]—that show circadian expression in the liver [31], which is supported by additional studies demonstrating that the CAR and AhR genes are clock-controlled [24,31,32]. Thus, there is a possibility that the molecular clock directly or indirectly, through xenobiotic receptors, also regulates the circadian expression of Abcc4.

The molecular clock that controls CP functions is regulated by sex hormones (SHs) [13]. Clock genes in CPs are differentially expressed between the sexes, and E2 modulates the expression of Per1, Per2, and Bmal1 [16,17]. There is also the possibility that Abcc4 is regulated by estrogens. A study with porcine endometrium explants showed that neither E2 nor P4 modulates Abcc4 expression [33]. Maher et al. corroborated these data, with no differential results shown between control, OVX, and OVX mice replaced with E2 [34]. However, there is a study reporting the involvement of estrogen receptors (ERs) in the expression of Abcc4, showing that the activation of ERs increases Abcc4 expression [35]. Dihydrotestosterone (DHT), on the other hand, upregulates Abcc4 in the LNCaP cell line [36,37]. However, in the present study, the rAbcc4 circadian pattern observed in intact female rats was lost in Sham and OVX females, as in the other transporters analyzed (rAbcg2 and rOat3). It is likely that the reason for this was the side effects of ketamine in Sham and OVX groups. In fact, there is experimental evidence that ketamine can influence circadian oscillations in different systems. For instance, ketamine caused a phase advance in the rhythms of rats’ locomotor activity when administered in the resting phase, whereas when administered during the active phase, a phase delay was caused [38]. In addition, at the molecular level, ketamine induces a phase shift in Bmal1 and Dbp expression. Thus, it is possible that the observed alterations in rAbcc4 circadian mRNA fluctuations in Sham and OVX female animals are a consequence of ketamine anesthesia because its expression is possibly controlled by the PARbZip transcription factors [24]. Thus, we cannot conclude that rAbcc4 rhythmicity in females is dependent on SHs.

Regarding Abcg2, Hamdan et al. demonstrated a circadian rhythm oscillation in the small intestine of mice, with peak expression occurring during the light phase. This circadian expression is thought to be dependent on the molecular clock since Abcg2 rhythmic expression is completely abolished in *Clock* mutant mice [39]. Later, in another study, it was also described that Abcg2 presented circadian expression in the mouse liver, and again, this expression was dependent on the molecular clock, as confirmed in a *Per1* and *Per2* double transgenic mouse [40]. Contrarily to the small intestine, peak expression in the liver was reported during the dark phase [40]. In our study, rAbcg2 was rhythmic only in intact females, with its peak expression in the first half of the dark phase. Like rAbcc4, rAbcg2 lost the circadian pattern in Sham and OVX females, and for the same reason, we cannot conclude that the rhythmic expression of rAbcg2 in the rats’ CPs is dependent on female SHs.

Regarding rOat3, we showed rhythmicity in the CPs of intact male and female rats but not in OVX or Sham-operated females. Our results are in line with a study that reported decreased expression of Oat3 in the kidneys of Bmal1 KO rats compared with controls, suggesting that Oat3 is a clock-controlled gene [41]. Overall, our results seem to show that the molecular clock is responsible for the rhythmicity of rAbcc1, rAbcc4, rAbcg2, and rOat3 in rat CPs. However, the reasons for the absence of circadian rhythmicity in Sham and OVX-operated female rats are not entirely understood, and further work needs to be carried out to elucidate the causes of these observations.

MTX is a substrate of several membrane transporters expressed in the CP, namely ABCB1, ABCC1, ABCC2, ABCC3, ABCC4, ABCC10, ABCG2, OAT1, OAT3, organic anion transporter polypeptide (OATP) 1C1, proton-coupled folate transporter (PCFT), peptide transporter (PEPT) 1, and reduced folate carrier (RFC) [19,42,43,44,45,46,47,48,49,50,51,52]. Furthermore, it is well-documented that ABCC1, ABCC4, and PCFT are expressed in the basolateral membrane of CPEC [20,52,53]; SLCO1C1 in the basolateral and in the apical membrane [54]; and ABCB1, ABCG2, and RFC are located in the apical membrane [42,53,55]. Folate receptor α, which mediates the transcytosis of MTX, is also located in the apical membrane of CPEC [56].

To explore the idea that the ABCC4 function varies according to the time of day, we studied the transport of MTX across the BCSFB using an in vitro uptake assay. Interestingly, we observed daily oscillations in FL–MTX concentrations in the three compartments (basolateral, apical, and intracellular), demonstrating that MTX is transported across the basal and the apical membranes of HIBCPP cells in a circadian way. This result correlates favorably with several authors who demonstrated the circadian rhythm on MTX pharmacokinetics in animal and human studies [57,58,59], supporting the idea of the effect of timing of drug administration with the pharmacokinetic parameters of MTX [60]. It is interesting to note that the peak observed in MTX transport in the basal compartment occurred around 20 h after synchronization, and the peak hAbcc4 mRNA expression in HIBCPP cells occurred around 10 h after synchronization. The 10 h lag observed between these two in vitro experiments using six timepoints is in line with the dynamic phases occurring from mRNA to protein expression. MTX secondary peaks were also detected in the apical and cellular compartments. As described above, MTX is a substrate for diverse CPs’ membrane transporters, and the differential expression of these transporters could modulate circadian activity [31]. The different timings of peak expression and, consequently, distinct peaks of activity might be a reason to justify the presence of secondary peaks in MTX uptake in both apical and cellular compartments.

To further explore the involvement of ABCC4 in the circadian transport of MTX across the BCSFB, we performed an in vitro uptake assay using an ABCC4 inhibitor. The rhythmicity of FL–MTX transport across the basal membrane was lost, whereas across the apical membrane and intracellularly, it was conserved. We believe that ABCC4 is, in part, responsible for the MTX circadian transportation across the basal membrane.

In a previous study, it was demonstrated that brain exposure to MTX through the BBB is coordinated by ABCC4 and ABCG2 transporters [61]. We thus examined whether the MTX transport depends on ABCG2. Curiously, we also observed a loss of rhythmicity in FL–MTX concentration in the basal compartment using an ABCG2 inhibitor. However, the FL–MTX concentration in the apical and intracellular compartments maintained the circadian pattern. Thus, it seems that the rhythmicity of FL–MTX transport across the basal membrane is not exclusively driven by ABCC4 because the inhibition of ABCG2 also impaired the rhythmicity of FL–MTX transport across the basal membrane. As put forward by Sane et al. [61], the evidence we found points to the idea of contributory roles of ABCC4 and ABCG2 to the circadian transport of MTX across the BCSFB.

Our results are a major step in understanding how the transport of therapeutic drugs is directly related to the circadian system. By understanding how the circadian system controls the circadian expression of ABC and SLC carriers and thus their circadian activity, we might become closer to improving SCN chronotherapeutic strategies. The timing of drug administration for the expected peak plasma levels must be in sync with the brain barrier’s peak permeability in order to achieve maximum therapeutic and reduced side effects, improving patients’ outcomes.

## 4. Material and Methods

### 4.1. Animals and Cell Line

In the present study, proestrus female and male Wistar rats at the age of 8–10 weeks were used. Wistar rats were divided in four experimental groups: intact males (*n* = 24), intact females (*n* = 24), ovariectomized (OVX; *n* = 24), and sham-operated female rats (Sham; *n* = 24). All animals were housed with standard laboratory chow and water ad libitum and were maintained under constant temperature and in 12 h light (07:00 h–19:00 h)/dark (19:00 h–07:00 h) cycles. Zeitgeber time (ZT) 0 was defined as lights on and ZT12 as lights off. Intact male and female rats were euthanized using a ketamine/xylazine anesthetic mixture. OVX and Sham rats were operated on under the administration of a ketamine/medetomidine solution, and two weeks after surgery, the rats were euthanized. The CPs from lateral ventricles were collected at four different time points (ZT1, ZT7, ZT13, and ZT19) and were immediately frozen in liquid nitrogen for quantitative real-time PCR analysis of ABC and SLC transporters.

All animal procedures followed the NIH guidelines and the European rules for care and handling of laboratory animals (Directive, 2010/63/EU).

The human epithelial CP papilloma (HIBCPP) cell line derived from a human malignant CP papilloma was kindly made available by C Schwerk [21]. These cells preserve the polygonal morphology and the phenotype of CP epithelial cells (CPEC). The HIBCPP cell line forms a functional epithelial barrier with high transepithelial electric resistance (TEER) values [21,62,63]. Therefore, it was used for functional studies.

### 4.2. HIBCPP Cell Culture

HIBCPP cells were seeded in 24-well plates with Dulbecco’s modified eagle medium (DMEM)/F12 (Gibco) supplemented with 10% Fetal Bovine Serum (FBS) (Life Technologies, Carlsbad, CA, USA), 1% penicillin/streptomycin (MP Biomedicals, Irvine, CA, USA), and 5 µg/mL insulin (Sigma-Aldrich, Algés, Portugal). The cells were kept in a humidified incubator at 37 °C and 5% CO_2_. One day after seeding, the medium was changed, and from that day, the medium was replaced every two days. After reaching 70% confluence, the cells were synchronized with 1% of dexamethasone for 2 h at 37 °C and 5% CO_2_.

To analyze the circadian pattern of hAbcc4 and hAbcg2 in the HIBCPP cell line, the cells were harvested for total RNA extraction at the following time points: 4, 8, 12, 16, 20, and 24 h after synchronization.

### 4.3. Quantitative Real-Time PCR (qPCR)

Total RNA was isolated from the rat CP and HIBCPP cells using TRIzol reagent (Grisp, Porto, Portugal) according to the manufacturer’s instructions. Total RNA purity and quantification were assessed by the measurement of the absorbances at 260 and 280 nanometers using a NanoPhotometer^TM^ (Implen, Munich, Germany). cDNA was synthesized using an NZY M-MuLV First-Strand synthesis kit (NZYTech, Lisbon, Portugal) according to the manufacturer’s protocol.

In animal experiments, quantitative real-time PCR (qPCR) was performed to assess the daily expression of rAbca1, rAbcb1, rAbcc1, rAbcc4, rAbcg2, rAbcg4, and rOat3. In the HIBCPP cells, qPCR was implemented to determine the daily expression of hAbcc4 and hAbcg2. qPCRs were performed on a CFX Connect^TM^ Real-Time PCR Detection System (Bio-Rad, Hercules, CA, USA) using an Xpert Fast SYBR 2X mastermix (Grisp, Porto, Portugal). qPCRs were carried out with an initial denaturation step at 95 °C for 3 min followed by 40 cycles of 95 °C for 5 s, 60 °C for 30 s, and 72 °C for 10 s. The amplification of all transcripts was validated by the profiles of melting curves. The relative expression of selected genes was calculated according to the ∆∆ct method [64]. The efficiency of all primers was previously tested with the following cDNA dilutions: 1, 1:2, 1:4, 1:8; and the sequences and amplicon sizes are listed in Table 3.

### 4.4. MTX Uptake Assay

**Figure 7:**
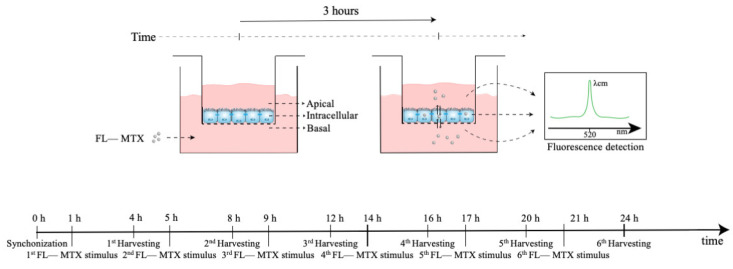
Scheme of the MTX uptake assay. At 0 h, the cells were synchronized using dexamethasone. At six different time points (1, 5, 9, 14, 17, and 21 h after synchronization) the transwell inserts were incubated with MTX in the basal compartment for 3 h. After the incubation, the MTX levels in the basal, intracellular, and apical compartments were assessed by fluorescence analysis.

The assessment of the MTX transport in HIBCPP cells was chosen to investigate whether the daily oscillations of Abcc4 expression seen in the CPs of intact male and female rats and the CP cell line would affect the transport of its substrate, MTX, across the BCSFB. Of interest, ABCC4 is also involved in the transport of several other compounds of pharmacological importance, such as antiviral, antibacterial, antihypertensive, and antineoplastic agents, as well as for the efflux of endogenous substrates, such as cAMP and prostaglandins [65]. HIBCPP cells were seeded in a transwell filter system (Figure 7) and used as an in vitro model of the BCSFB. For this, the HIBCPP cells were seeded in the apical compartment of cell culture inserts (pore diameter 0.4 µm and insert area 0.33 cm^2^; VWR, Alfragide, Portugal) at a density of 1.5 × 10^5^ cells per insert in DMEM/F12 supplemented with 10% FBS, 5 µM/mL insulin, and 1% penicillin/streptomycin. The culture medium was added to the basal compartment only two days after seeding and, from that day, the medium was changed every two days. The paracellular permeability was assessed every day by the measurement of TEER values using an Epithelial Volt/Ohm Meter (WPI, Sarasota, FL, USA). On the fourth day of culture, TEER values reached 300 Ω·cm^2^, and then the medium was changed and maintained with 1% FBS. On the seventh day of culture, the cells were synchronized with 1% dexamethasone for 2 h at 37 °C and 5% CO_2_. To verify the rhythmicity of MTX transport across BCSFB, after the synchronization and before the incubation with the substrate, the cells were washed three times and preincubated with Krebs–Ringer buffer (KRB). Next, the cells were incubated with MTX conjugated with fluorescein (FL–MTX; Biotium, Fremont, CA, USA) for 3 h, with the start of incubation at six different time points (1, 5, 9, 14, 17, and 21 h after synchronization) (Figure 7). After the 3 h incubation with the substrate, the apical and basal mediums were collected and pipetted onto a black plate. The remaining cells were washed three times with KRB and lysed by incubation with a Triton X-100 1% solution for 30 min at 37 °C. After the incubation, the lysis solution was homogenized and pipetted onto the black plate. The FL–MTX concentration was then determined by fluorescence reading using a SpectraMax Gemini spectrofluorometer (Molecular Devices, San José, CA, USA) at an excitation wavelength of 490 nm and an emission wavelength of 520 nm. Next, to explore the involvement of ABCC4 in the circadian rhythmicity of FL–MTX transport across the BCSFB, an additional assay was performed with the inhibitor of ABCC4 (applied in the basal compartment) ceefourin1 (5 µM; Tocris, Bristol, UK). Finally, because ABCG2 was also described as an important apical transporter in limiting the distribution of MTX, a similar assay was performed using an ABCG2 inhibitor (Ko143 100 nm; Tebu-bio, Lisbon, Portugal).

### 4.5. Statistical Analysis

The rhythmicity in the mRNA expression of CP transporters and the FL–MTX concentration in the three compartments (apical, basal, and intracellular) were analyzed by a harmonic regression method with an assumed period of 24 h and alpha set at 0.05 using the CircWave v1.4 software (Dr. Roelof A. Hut, Groningen, Netherlands) [66,67]. Statistically significant rhythms were considered when *p* < 0.05. Note that in the presence of rhythmicity, CirWave’s output was a sine wave and provided a significant *p*-value. When no rhythmic pattern was detected, the output was a straight line, and no *p*-value was given. Points and full bars represent the mean, and error bars represent the standard error of the mean (±SEM).

## 5. Conclusions

Brain barriers are a major hurdle in brain’s pharmacotherapy. Timing drug administration in order to synchronize plasma peak concentrations with maximum brain barrier permeability is a way to overcome this obstacle. We have proven that membrane transporters present a circadian rhythmic expression in the CP of rats. MTX transport across an in vitro model of the BCSFB was also found to be rhythmic and directly dependent of the ABCC4 circadian rhythmicity. This is a clear indication of the influence of chronobiology in the transport of drugs across the BCSFB. Thus, in the future, a better understanding of how the circadian rhythms influence membrane transporters activity might be a staple in personalized brain pharmacotherapy.

## Figures and Tables

**Table 1:** Significance (*p*-value) and center of gravity (COG) values for rAbcc1, rAbcc4, rAbcg2, and rOat3, as determined by CircWave analysis.

rAbcc1	Intact males	*p*-value = 0.0086 COG = 4.65
rAbcc4	Intact females	*p*-value = 0.0080 COG = 14.20
	Intact males	*p*-value = 0.0429 COG = 7.43
rAbcg2	Intact females	*p*-value = 0.0447 COG = 13.66
rOat3	Intact females	*p*-value = 0.0079 COG = 17.27
	Intact males	*p*-value = 0.0312 COG = 18.80

rAbca1, rAbcb1, rAbcc1, rAbcc4, rAbcg2, rAbcg4, and rOat3 in both Sham and OVX groups did not show significant daily oscillations.

**Table 2:** Significance (*p*-value) and center of gravity (COG) values for hAbcc4 and hAbcg2 determined by CircWave analysis.

hAbcc4	*p*-value = 0.0068 COG = 9.91
hAbcg2	*p*-value = 0.0001 COG = 10.74

**Table 3:** Primers and amplicons sizes used for real-time quantitative PCR.

Gene	Primer Sequence (5′–3′)	Amplicon Size (bp)
hAbcc4	FW: TGTGGCTTTGAACACAGCGTA	105
	RV: CCAGCACACTGAACGTGATAA	
hAbcg2	FW: ACGAACGGATTAACAGGGTCA	93
	RV: CTCCAGACACACCACGGAT	
hGapdh *	FW: ATGGGGAAGGTGAAGGTCG	108
	RV: GGGGTCATTGATGGCAACAATA	
rAbca1	FW: CGGCGGAGTAGAAAGGGTTT	84
	RV: CACGATCAGGCTGAAGACCAG	
rAbcb1	FW: AAGGGGCTACAGGGTCTAGG	100
	RV: AGTGTCAATTGCCAGCCGTA	
rAbcc1	FW: TTCATATCTGCTTCGTCACCG	60
	RV: CGTAAACAGCACCCACCACAGC	
rAbcc4	FW: TTCCCCTTCGACCTTATCCT	124
	RV: TAGGCAGCTGTTGTCAGTGG	
rAbcg2	FW: GGCCTGGACAAAGTAGCAGA	137
	RV: GTTGTGGGCTCATCCAGGAA	
rAbcg4	FW: ATGGCTGATGTACCCTTCCAGGTT	155
	RV: ATCAAGAGTCCCAAAGACTGGGCA	
rOat3	FW: GAGGACCTGTGATTGGAGAACTG	82
	RV: CTG GCT GCC AGC ATG AGA TA	
rCycA *	FW: CAAGACTGAGTGGCTGGATGG	163
	RV: GCCCGCAAGTCAAAGAAATTAGAG	

* Were used as reference genes.
